# Could we use a lower dose of rituximab to treat rheumatoid arthritis in clinical practice: pros and cons?

**DOI:** 10.1186/s13075-016-1022-1

**Published:** 2016-06-02

**Authors:** Gianfranco Ferraccioli, Barbara Tolusso, Elisa Gremese

**Affiliations:** Institute of Rheumatology, School of Medicine, Catholic University of the Sacred Heart, CIC-Via Moscati 31, Rome, 00168 Italy; Institute of Rheumatology, Fondazione Policlinico Gemelli, Catholic University of the Sacred Heart, CIC-Via Moscati 31, Rome, 00168 Italy

**Keywords:** Rituximab, Low dose, High dose, Synovial tissue B cell depletion, Peripheral B cell depletion

## Abstract

The CERERRA database provides evidence that low-dose rituximab performs as well as the conventional dose in the real world, thus highlighting the possible pharmacoeconomic impact. In clinical trials, it has been shown that rituximab 500 mg twice, performs as well as 1 g twice, 2 weeks apart, in terms of the American College of Rheumatology (ACR)20 and ACR50, but not the ACR70. The choice should always be made after considering that the IMAGE trial has demonstrated similar radiographic progression after the first 6 months, but with less control, with low-dose rituximab in the first 6 months. A possible alternative can be hypothesized.

## Editorial

One of the personalized approaches to rheumatoid arthritis (RA) has been obtained with rituximab. Since inception it has been observed that RA patients treated with rituximab, positive for rheumatoid factor (RF) or anti-cyclic citrullinated peptide autoantibodies (ACPA), obtained more clinical benefits than seronegative patients [1–3].

The rationale for using B-cell depletion was based on the idea that RA is the consequence of a failure of B-cell death in the synovium [4].

Whether peripheral blood B-cell depletion means the same as tissue depletion is debatable. In one patient with idiopathic thrombocytopenic purpura, no B cells were seen in the peripheral blood or in the bone marrow or the spleen 3 months after the final rituximab infusion (375 mg/m^2^ weekly for 4 weeks) [5]. Looking more closely at RA, however, there is synovial tissue evidence that, after 4 weeks following rituximab infusion (1 g twice, 2 weeks apart), all of 17 biopsied patients had peripheral B-cell depletion, yet only in three patients had B cells disappeared from the synovial tissue. Therefore rituximab was only partially effective in depleting B cells in the majority of the synovial tissues [6]. Whether the lack of B-cell depletion in the tissue depends on the interval between rituximab infusion and tissue analysis being too short, or because tissue depletion is really variable, even more so at different dosages, is not known. Accordingly, we need to rely almost exclusively on trials or registries to select the doses.

In clinical trials the 500 mg twice dose gave similar results to the 1 g twice dose when measuring the American College of Rheumatology 20 % improvement (ACR20) and ACR50 responses, but not for the major outcomes of ACR70 and Good EULAR response [7]. In the MIRROR trial, the high dose performed better [8]. In the study by Chatzidionysiou et al. [1] the authors examined the clinical response to two doses of rituximab (low dose (LD), 500 mg twice; and conventional dose (SD), 1 g twice) in more than 2800 patients with RA from 12 countries. The two cohorts differ at baseline for several factors, but the three most relevant for the outcome after rituximab were the number of patients (LD *n* = 248 vs CD *n* = 2625), disease activity (DAS-ESR28) and health assessment questionnaire (HAQ) levels being lower with the LD schedule, and in the percentage of patients who received previous tumor necrosis factor (TNF) blockers being higher with the LD schedule [1].

It is well known that the lower the level of disease activity, the higher is the percentage of success in terms of low-disease activity (LDA) or disease remission (DR). Moreover, the chance of obtaining LDA or DR is higher for the first biologic than for the second or third biologic [9]. Yet, in this study the delta of improvement was significantly better in the CD cohort, even though the trend suggested that, the longer the observation period, the lower the difference between the two dosages. Overall the main conclusion was that there was “evidence about the lack of any striking difference and perhaps no clinically significant difference between two different doses of rituximab used in clinical practice”.

The Authors recognize that the lack of radiographic data do not allow us to definitely state that the doses are similarly effective from all viewpoints. In fact, the IMAGE trial [10] showed a lower effectiveness of the LD using radiographic damage in the first 6 months, this being similar to the control of structural progression in the second 6 months.

In the study of Chatzidyonisiou et al. improvements were already present after 3 months, suggesting that a tight control of the outcomes following a treat-to-target strategy (major clinical assessment every 3 months) is possible in real life at both dosages. Given that similar results were seen after 3 months, as well as after 6 months, in a treat-to-target strategy the CD would very likely offer much more certainty of a result.

In conclusion, this study shows that in active, long standing RA with functional impairment, a LD schedule of rituximab gives 6-month follow-up clinical results similar to those obtained with CD, but the data have to be interpreted in the absence of radiographic analysis. The lack of a long-term assessment makes any possible choice in terms of clinical practice difficult because we have no idea when the LD schedule would lead us to re-treat patients when compared to the CD.

**Fig. 1:**
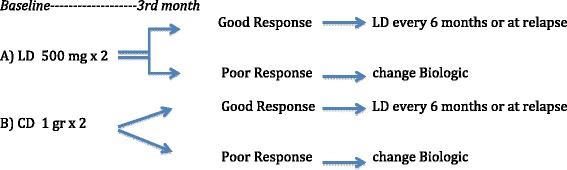
Rituximab in rheumatoid arthritis patients with moderate (**a**) or high (**b**) disease activity at baseline. A possible pragmatic approach following a treat-to-target strategy in the real world. The low-dose (*LD*) schedule for rituximab is likely appropriate in those with moderate disease activity at baseline, with the conventional dose (*CD*) in those with high disease activity at baseline. More data are needed on a flexible schedule to be used according to the effective B-cell depletion

The most important message relies on the possible pharmacoeconomic consequences. The real clue, not proven by the data available here however, could be the possible third choice, demonstrated to be realistic by Mariette et al. [11]: first infusion with the CD schedule and, in cases with good response (DR or LDA), the LD schedule for the re-treatments. This would really allow us to save a lot of money (Fig. 1).

## Abbreviations

ACR, American College Rheumatology percentage of improvement; CD, conventional dose; DR, disease remission; LD, low dose; LDA, low disease activity; RA, rheumatoid arthritis.
